# Editorial: Combat sports and wellbeing: advancing health and inclusion in athletes and practitioners

**DOI:** 10.3389/fpsyg.2025.1715508

**Published:** 2025-10-16

**Authors:** Simone Ciaccioni, Flavia Guidotti, Elena Pocecco, Nemanja Stankovic, Pascal Izzicupo

**Affiliations:** ^1^Department of Education and Sport Sciences, Pegaso Telematic University, Naples, Italy; ^2^Department of Movement, Human and Health Sciences, University of Rome “Foro Italico”, Rome, Italy; ^3^Department of Human Sciences and Promotion of Quality of Life, University of Rome “San Raffaele”, Rome, Italy; ^4^Department of Sport Science, University of Innsbruck, Innsbruck, Austria; ^5^Faculty of Sport and Physical Education, University of Niš, Niš, Serbia; ^6^Department of Medicine and Aging Sciences, University of Chieti-Pescara “Gabriele D'Annunzio”, Pescara, Italy

**Keywords:** Paralympic and Olympic combat sports, martial arts, mental health, children with disability, adolescents, disability, adaptive sports, sport psychology

## Introduction

Combat sports (CS) and martial arts (MA) are among the oldest forms of organized human activity, combining technical skills, discipline, and competition with cultural, philosophical, and social significance. In contemporary times, they have been framed through the lens of athletic excellence, tactical performance, and competitive outcomes. Yet in the past two decades, the scientific community has increasingly recognized their broader role as tools for promotion of health, psychological resilience, and social inclusion. This evolving paradigm shift has been reinforced by a growing body of empirical evidence, demonstrating that CS and MA can be viewed not only as competitive practices but also as interventions for improving wellbeing across the lifespan and in diverse populations.

The present Research Topic, *Combat Sports and Wellbeing: Advancing Health and Inclusion in Athletes and Practitioners*, gathers 13 peer-reviewed contributions spanning original research, systematic and narrative reviews, mini-reviews, and opinion papers. Collectively, these works highlight this research area from different perspectives and underscore how CS and MA can be leveraged to enhance quality of life, psychological resilience, and social cohesion. Hereinafter, we synthesize these contributions organizing them into four major thematic domains: psychological resilience, physical and mental health outcomes, inclusion and adaptive practice, and innovation and contemporary applications. Finally, we reflected on the translational and practical implications of this body of work and highlighted emerging directions for future research in this field.

## Psychological resilience

A defining thread across multiple studies in this Research Topic is the consistent evidence that CS and MA foster psychological resilience, self-regulation, and emotional competence. These qualities are crucial not only for athletic performance but also for coping with life stressors, recovering from adversity, and cultivating positive mental health, and even more important for young people, who are developing their personality.

In a cross-cultural context, Spantios et al. examined psychometric properties among kendo practitioners compared to non-practitioners by means of an online survey. By identifying psychological and cultural profiles, their work contributes to the understanding of how CS and MA shape resilience and wellbeing in various sociocultural environments.

Women's empowerment and safety emerged as a specific line of inquiry. Pekel et al. as corrected in Frontiers Production Office investigated the potential of martial arts-based self-defense training to bolster psychological strength among women. Major findings highlighted how structured martial arts programs can cultivate self-confidence, autonomy, and resilience, thereby contributing to gender-specific approaches to health and security.

Intervention-based studies reinforced these findings in specific CS and MA disciplines. Yikilmaz et al. reported that taekwondo training enhanced women's quality of life, improved self-defense competence, and strengthened psychological resilience. Similarly, Köroglu et al. showed that judo practice facilitated emotional expression, self-control, and resilience, underlining the psychosocial benefits of structured training environments.

Predoiu et al. examined resilience of competitive athletes practicing Olympic combat sports, revealing resilience as a defining psychological resource across disciplines. This work bridges the gap between amateur participation and elite performance, highlighting resilience as a transferable outcome of CS and MA engagement, regardless of competitive context.

Altogether, these contributions affirm that CS and MA offer a unique environment for cultivating resilience, balancing exposure to controlled stressors with the development of adaptive coping mechanisms.

## Physical and mental health

Another substantial cluster of studies focused on health-related outcomes of CS and MA practice, spanning physiological, psychological, and quality-of-life dimensions.

Sahin et al. demonstrated that Muay Thai training improved quality of life, self-control, and love of life, reinforcing the knowledge that CS and MA can promote positive affective and cognitive states in addition to physical fitness.

From a physiological stress perspective, Okudan et al. investigated the impact of body weight-loss practices in wrestlers. Their findings underlined the stress that rapid weight reduction imposes, implying both physical and psychological potential harmful responses. This study raises important concerns for athlete safety highlighting the need for evidence-based guidelines for safe weight-loss strategies to mitigate health risks.

The cardiovascular system was the focus of the paper by Rossi et al., who conducted a narrative review of judo. By synthesizing research on acute and chronic cardiovascular adaptations, they provided an integrative overview of how judo training impacts heart health, aerobic capacity, and systemic responses. Such research informs both athletic conditioning and clinical exercise applications, suggesting potential translational benefits for cardiovascular health promotion.

Collectively, these studies broaden the scope of CS and MA research, demonstrating that the impact of such activities extends well beyond competitive performance into areas of health promotion, risk management, and prevention of diseases.

## Inclusion, diversity, and special populations

A defining feature of this Research Topic is the emphasis on CS and MA as inclusive practices, accessible to individuals with diverse abilities, backgrounds, and health conditions.

Lee et al. offered a systematic review of controlled trials investigating Olympic combat sports for children and adolescents with disabilities. The review highlighted promising evidence for improvements in mental health, psychosocial outcomes, and inclusion, though it also called for higher methodological rigor and larger trials in future research.

Complementing this evidence, Nerozzi et al. presented a mini-review on adaptive judo for individuals with neuropathy, showing how tailored practice can enhance motor skills, balance, and quality of life. Their work illustrates the adaptability of judo in therapeutic and rehabilitative contexts, reinforcing the idea that CS and MA can be inclusive for individuals with different functional abilities.

The conceptual framing of this inclusive agenda was provided by Ciaccioni et al., whose opinion paper showed the potential of CS and MA in advancing health and inclusion across heterogeneous populations. This perspective article synthesized notions from evidence and practice, calling for strategic integration of CS and MA into broader health promotion and educational initiatives.

By bringing together evidence- and eminence-based perspectives, these contributions show that CS and MA can promote social inclusion of different special populations, e.g., children or adults with special needs as well as clinical populations.

## Innovation and contemporary applications

In addition to traditional settings, several contributions explored innovative applications of CS and MA. Oh et al. examined the impact of intramural martial arts participation on the wellbeing of international students, testing a sequential mediation model. Findings indicated that participation significantly enhanced wellbeing, both directly and indirectly, through the pathway of positive emotions fostering stress relief, which in turn promoted ego-resilience.

Technological innovation was addressed by Li et al., who conducted a mini-review on the applications of virtual reality in combat sports. The authors outlined how immersive technologies have the potential to enhance the acquisition of new skills, improve physical fitness, provide safe training environments, and reduce the psychological pressure of facing real opponents. This work opens new avenues for the digital transformation of training in specific CS and MA and seems to be particularly relevant in contexts where in-person participation is limited.

In summary, these contributions underline the evolving adaptability of CS and MA, which have the potential of extending their impact into new cultural and educational domains as well as to make use of innovative technologies.

## Toward a translational agenda

**Figure 1:**
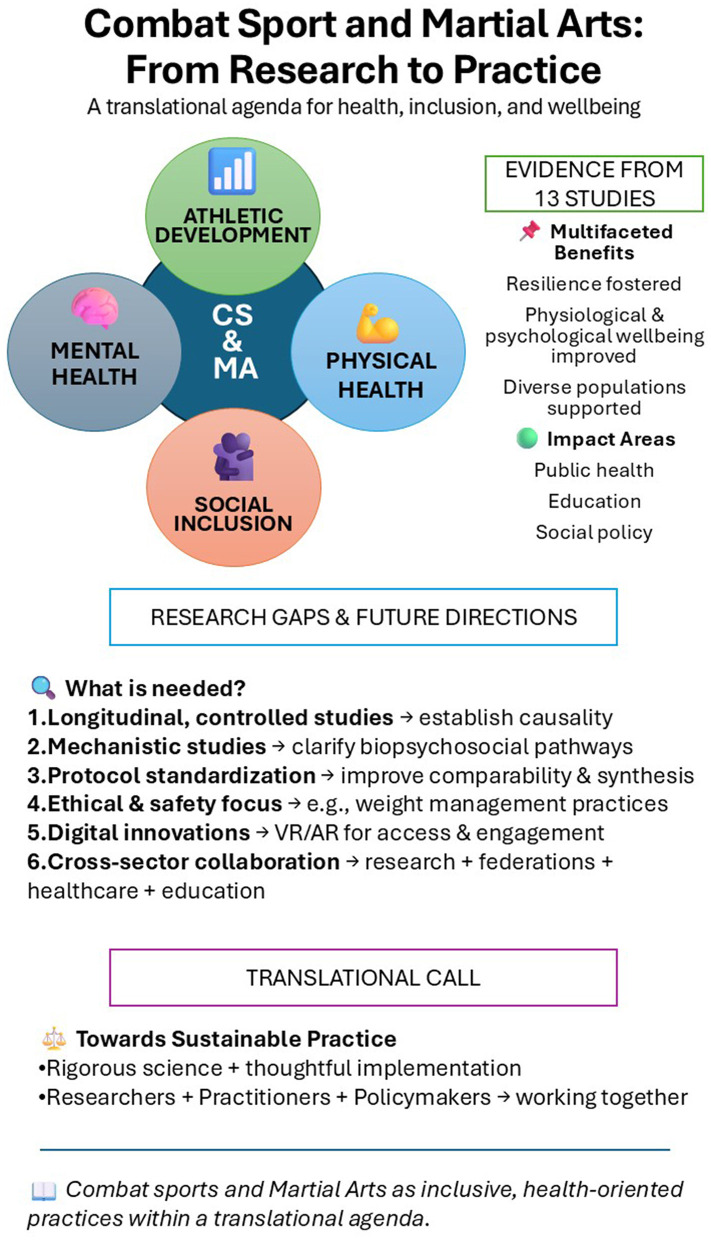
AR, augmented reality; CS, combat sports; MA, martial arts; VR, virtual reality.

The 13 studies belonging to this Research Topic converge to the conclusion that CS and MA represent multifaceted practices that simultaneously serve athletic, physical as well as psychological health-promoting, social and inclusive functions. By fostering resilience, improving physiological and psychological health, and supporting diverse populations, they prove to be valuable resources for public health, education, and social policy (Figure 1).

However, important challenges still persist. Therefore, future research should address several critical gaps:

- Longitudinal, controlled designs are required to establish causal relationships between CS and MA participation and health or wellbeing outcomes.
- Mechanistic studies should elucidate the biopsychosocial pathways whereby CS and MA exert their effects.
- Standardization of protocols across studies has the potential of improving comparability and synthesis, enhancing the robustness of evidence.
- Ethical and safety considerations, particularly regarding weight-management practices, should remain a priority in terms of athletes' health preservation.
- Digital innovations, including virtual and augmented reality, should be further explored as tools to broaden access and engagement.
- Cross-sector collaborations between research institutions, sports federations, healthcare providers, and educational bodies could translate evidence-based knowledge into sustainable practice.

Positioning CS and MA within a translational agenda thus requires both rigorous scientific studies and thoughtful implementation. Researchers, practitioners, and policymakers should therefore work together to harness their potential as inclusive, health-oriented practices.

## Conclusion

This Research Topic shows that CS and MA are not merely performance-driven disciplines but powerful vehicles for improving resilience, physical and mental health, and inclusion. By integrating evidence-based knowledge across psychological, physiological, and social dimensions, these contributions collectively redefine CS and MA as holistic practices with relevance that extends far beyond the arena.

As the CS and MA field continues to develop, future efforts should emphasize methodological rigor in scientific research, interdisciplinary collaborations, and innovative applications. Whether by fostering women's empowerment, enhancing cardiovascular health, advancing students' wellbeing and inclusion, or tailoring practice to individuals with special needs, CS and MA provide a versatile platform for generating positive impact at both individual and community levels.

By bridging tradition and innovation, competition and compassion, CS and MA can meaningfully contribute to contemporary health agendas and inspire new generations of scholars, practitioners, coaches, and policymakers to leverage their transformative potential.

